# Supplementary material for: Spectinamide MBX-4888A exhibits favorable lesion and tissue distribution and promotes treatment shortening in advanced murine models of tuberculosis
Source: Antimicrob Agents Chemother. 2024 Sep 30;68(11):e00716-24. doi: 10.1128/aac.00716-24 (PMC11539231; doi:10.1128/aac.00716-24)
Supplement: Supplemental material — Tables S1 to S5; Figures S1 to S3. [file aac.00716-24-s0001.docx]

**Title: Spectinamide MBX-4888A exhibits favorable lesion and tissue distribution and promotes treatment shortening in advanced murine models of tuberculosis**

**Authors**

Allison A. Bauman^a^, Jansy P. Sarathy^b^, Firat Kaya^b^, Lisa M. Massoudi^a^, Michael S. Scherman^a^, Courtney Hastings^a^, Jiuyu Liu^c^, Min Xie^b^, Elizabeth J. Brooks^a^, Michelle E. Ramey^a^, Isabelle L. Jones^a^, Noalani D. Benedict^a^, Madelyn R. Maclaughlin^a^, Jake A. Miller-Dawson^a^, Samanthi L. Waidyarachchi^d^, Michelle M. Butler^d^, Terry L. Bowlin^d^, Matthew D. Zimmerman^b^, Anne J. Lenaerts^a^, Bernd Meibohm^e^, Mercedes Gonzalez-Juarrero^b^, Michael A. Lyons^a^, Véronique Dartois^b^, Richard E. Lee^c^, and Gregory T. Robertson^a,f^

**Affiliation:**

^a^Mycobacteria Research Laboratories, Department of Microbiology, Immunology and Pathology, Colorado State University, Fort Collins, Colorado, USA.

^b^Center for Discovery and Innovation, Hackensack Meridian School of Medicine, Nutley, New Jersey, USA.

^c^Department of Chemical Biology & Therapeutics, St. Jude Children’s Research Hospital, Memphis, Tennessee, USA.

^d^Microbiotix, Inc., Worcester, MA 01605, USA

^e^College of Pharmacy, Department of Pharmaceutical Sciences, University of Tennessee Health Sciences Center, Memphis, Tennessee, USA.

^f^Corresponding Author: Gregory.Robertson@colostate.edu

**Keywords:** Tuberculosis, relapse, caseum, drug combinations, C3HeB/FeJ

**Running Title:** Diverse mouse pathology and regimen performance **Suppl. Table 1:** P values using the Fisher's exact test for the proportions of mice relapsing 3-months (12 weeks) after 2HRZE/HR or 2HRZE/HR plus MBX-4888A for the indicated treatment time (in months).

| **BALB/c arm:**  **Regimen/Treatment duration in months** | | **HRZE/HR** | | **HRZE/HR+4888A** | |
| --- | --- | --- | --- | --- | --- |
|  |  | **M3+3** | **M4+3** | **M3+3** | **M4+3** |
| **HRZE/HR** | **M3+3** | **NA** |  |  |  |
|  | **M4+3** | 0.2063 | **NA** |  |  |
| **HRZE/HR+4888A** | **M3+3** | 0.0996 | 0.6546 | **NA** |  |
|  | **M4+3** | <0.0001 | 0.0018 | 0.0092 | **NA** |

| **C3HeB/FeJ arm:**  **Regimen/Treatment duration in months** | | **HRZE/HR** | | | **HRZE/HR+4888A** | | |
| --- | --- | --- | --- | --- | --- | --- | --- |
|  |  | **M3+3** | **M4+3** | **M5+3** | **M3+3** | **M4+3** | **M5+3** |
| **HRZE/HR** | **M3+3** | **NA** |  |  |  |  |  |
|  | **M4+3** | 0.0996 | **NA** |  |  |  |  |
|  | **M5+3** | 0.0007 | 0.1394 | **NA** |  |  |  |
| **HRZE/HR+4888A** | **M3+3** | >0.9999 | 0.3295 | 0.0052 | **NA** |  |  |
|  | **M4+3** | 0.0007 | 0.1394 | >0.9999 | 0.0052 | **NA** |  |
|  | **M5+3** | <0.0001 | 0.0005 | 0.0801 | <0.0001 | 0.0801 | **NA** |

**Suppl. Table 2.** Percent (%) lung involvement per lesion type at the start of treatment by LIRA.

| **Mouse** | **Percent (%) lung involvement per lesion type at the start of treatment** | | | | | | **# of Type Is** |
| --- | --- | --- | --- | --- | --- | --- | --- |
|  | **Healthy Tissue** | **Type I - Caseum** | **Type II** | **Type III** | **Type I - Rim** | **Unknown/Misc** |  |
| Mouse K | 75.7 | 3.1 | 0.0 | 17.2 | 4.0 | 0.0 | 2 |
| Mouse L | 70.8 | 0.3 | 0.0 | 28.4 | 0.5 | 0.0 | 1 |
| Mouse M | 83.8 | 0.0 | 0.0 | 16.2 | 0.0 | 0.0 | 0 |
| Mouse N | 83.2 | 0.0 | 0.0 | 16.8 | 0.0 | 0.0 | 0 |
| **Average** | **78.5** | **0.8** | **0.0** | **19.5** | **1.1** | **0.0** | **0.8** |

**Suppl. Table 3.** Percent (%) lung involvement per lesion type after 2-months of HRZE treatment by LIRA.

| **Mouse** | **Percent (%) lung involvement per lesion type after 2-months of HRZE** | | | | | | **# of Type Is** |
| --- | --- | --- | --- | --- | --- | --- | --- |
|  | **Healthy Tissue** | **Type I - Caseum** | **Type II** | **Type III** | **Type I - Rim** | **Unknown/Misc** |  |
| Mouse K | 91.4 | 0.0 | 0.0 | 8.6 | 0.0 | 0.0 | 0 |
| Mouse L | 91.5 | 2.2 | 0.0 | 4.4 | 1.9 | 0.0 | 1 |
| Mouse M | 95.9 | 0.0 | 0.0 | 4.1 | 0.0 | 0.0 | 0 |
| Mouse N | 92.7 | 0.4 | 0.0 | 5.7 | 1.2 | 0.0 | 1 |
| **Average** | **92.9** | **0.7** | **0.0** | **5.7** | **0.8** | **0.0** | **0.5** |

**Suppl. Table 4.** Rifampin (RIF), pyrazinamide (PZA) and MBX-4888A drug concentrations evaluated in the *ex vivo* caseum bactericidal activity assay.

|  | Drug concentration in µM | | |
| --- | --- | --- | --- |
| Well # | RIF | PZA | MBX-4888A |
| 1 | 0.016 | 0.313 | 0.039 |
| 2 | 0.063 | 1.250 | 0.156 |
| 3 | 0.25 | 5 | 0.625 |
| 4 | 1 | 20 | 2.5 |
| 5 | 4 | 80 | 10 |
| 6 | 16 | 320 | 40 |
| 7 | 64 | 1280 | 160 |

**Suppl. Figure 1**. Dose-response curves for spectinamide MBX-4888A in the *ex vivo* caseum bactericidal activity assay versus *Mycobacterium tuberculosis* HN878.

**Suppl. Figure 2**. Dose-response curves for rifampin (RIF) and pyrazinamide (PZA) in the *ex vivo* caseum bactericidal activity assay versus *Mycobacterium tuberculosis* HN878.

**Suppl. Figure 3**. Dose-response curves for MBX-4888A, rifampin (RIF) and pyrazinamide (PZA) in the *ex vivo* caseum bactericidal activity assay.

**Suppl. Table 5.** Dose-response parameters for rifampin (RIF), pyrazinamide (PZA) and MBX-4888A drug concentrations in the *ex vivo* caseum bactericidal activity assay.

| Drug | E_max_ | EC_50_, 4888A (µM) | EC_50_, RIF (µM) | EC_50_, PZA (µM) |
| --- | --- | --- | --- | --- |
| MBX-4888A | 0.7 | 40 | - | - |
| RIF+PZA | 2.9 | - | 20 | 400 |
| MBX-4888A+RIF+PZA | 2.7 | 10 | 5 | 90 |

**Uncertainty in the parameter estimates - with p-value related metrics - indicating the degree to which the curves fit the data.**

|  | Formula: Y ~ (Emax * conc)/(conc + EC50) | | |  |  |
| --- | --- | --- | --- | --- | --- |
|  | Parameters: |  |  |  |  |
| **MBX-4888A** | Estimate | Std. Error | t value | Pr(>\|t\|) |  |
| Emax | 0.6637 | 0.09443 | 7.029 | 0.000206 | *** |
| EC50 | 37.15929 | 20.8606 | 1.781 | 0.118068 |  |
|  | --- |  |  |  |  |
|  | Signif. codes: 0 *** 0.001 ** 0.01 * 0.05 . 0.1 1 | | |  |  |
|  | Residual standard error: 0.08938 on 7 degrees of freedom | | | |  |
|  |  |  |  |  |  |
|  | Formula: Y ~ (Emax * conc)/(conc + EC50) | | |  |  |
|  | Parameters: |  |  |  |  |
| **RIF+PZA** | Estimate | Std. Error | t value | Pr(>\|t\|) |  |
| Emax | 2.8918 | 0.3474 | 8.324 | 0.000409 | *** |
| EC50 | 401.2235 | 127.5013 | 3.147 | 0.02547 | * |
|  | --- |  |  |  |  |
|  | Signif. codes: 0 *** 0.001 ** 0.01 * 0.05 . 0.1 1 | | |  |  |
|  | Residual standard error: 0.1389 on 5 degrees of freedom | | | |  |
|  |  |  |  |  |  |
|  | Formula: Y ~ (Emax * conc)/(conc + EC50) | | |  |  |
|  | Parameters: |  |  |  |  |
| **MBX-4888A+RIF+PZA** | Estimate | Std. Error | t value | Pr(>\|t\|) |  |
| Emax | 2.7377 | 0.2092 | 13.087 | 4.65E-05 | *** |
| EC50 | 92.8766 | 26.9056 | 3.452 | 0.0182 | * |
|  | --- |  |  |  |  |
|  | Signif. codes: 0 *** 0.001 ** 0.01 * 0.05 . 0.1 1 | | |  |  |
|  | Residual standard error: 0.184 on 5 degrees of freedom | | | |  |
